# Ce-Doped Graphitic Carbon Nitride Derived from Metal Organic Frameworks as a Visible Light-Responsive Photocatalyst for H_2_ Production

**DOI:** 10.3390/nano9111539

**Published:** 2019-10-30

**Authors:** Liangjing Zhang, Zhengyuan Jin, Shaolong Huang, Yiyue Zhang, Mei Zhang, Yu-Jia Zeng, Shuangchen Ruan

**Affiliations:** 1 Center for Advanced Material Diagnostic Technology, Shenzhen Technology University, Shenzhen 518118, China; zhangliangjing@sztu.edu.cn; 2 Shenzhen Key Laboratory of Laser Engineering, College of Physics and Optoelectronic Engineering, Shenzhen University, Shenzhen 518060, China; zhengyuan@szu.edu.cn (Z.J.); nkhsl3313@163.com (S.H.); yiyuezhang92@163.com (Y.Z.); 3 School of Materials Science and Engineering, Beijing Institute of Fashion Technology, Beijing 100029, China; zhangmei7115@163.com

**Keywords:** metal-organic framework, carbon nitride, H_2_ evolution, visible light-responsive

## Abstract

Novel fibrous graphitic carbon nitride (g-C_3_N_4_) derivatives prepared from metal organic frameworks (MOFs) were doped with Ce^3+^ (Ce-C_3_N_4_) as photocatalytic materials. Ce-C_3_N_4_ was characterized using various techniques, revealing its high specific surface area, excellent photocatalytic activity, and stability for H_2_ evolution under visible light irradiation. The fluorine modified samples show superior photocatalytic activity under visible light irradiation, which is due to the presence of more active sites and enhanced absorption of solar energy. This work provides a new synthetic route for MOF-derived g-C_3_N_4_ that can be doped with different metal ions. The fluorine modified Ce-C_3_N_4_ is an efficient photocatalyst with potential for many applications related to energy and the environment.

## 1. Introduction

Hydrogen is a promising clean and efficient alternative energy source that can be used to meet environmental and social energy challenges. One emerging method to produce hydrogen is through harnessing the energy of visible light from sunlight to split water and thus obtain hydrogen as a clean fuel [1,2,3]. To this end, careful selection of raw materials and synthetic methods to develop new efficient and stable photocatalysts has increasingly become the focus of attention in this field.

Graphitic carbon nitride (g-C_3_N_4_) is an inexpensive visible light photocatalyst with excellent optical, thermal, and electrical properties that has attracted considerable research interest, resulting in the development of numerous modified photocatalysts [4,5,6,7]. In order to improve the photocatalytic performance of g-C_3_N_4_, a variety of methods have been adopted to overcome its shortcomings (e.g., insufficient sunlight absorption, small surface area, and high photoinduced charge carrier recombination). These methods include exfoliation by ultrasonic treatment [8], heating method [9], hard template method [10], composite heterojunction [11,12,13,14], and metal [15,16,17] and non-metal doping [18,19,20,21]. Reducing the layer numbers of g-C_3_N_4_ through peeling or thermal corrosion changes the morphology and structure, resulting in more active sites on the surface and shortened path for carrier diffusion. Doping g-C_3_N_4_ with different metal ions or non-metals can alter the electronic structure and tune the band gap, which improve the light absorption, promote the charge separation and transportation, and extend the charge carrier lifetime [22]. Morphology control is considered to be another effective way to increase the surface property and catalytic activity. There are some reports on the regulation of g-C_3_N_4_ morphology, including nanorods [23,24], nanofibers [25], nanotubes [26], nanobelts [27], nanowires [28], and three-dimensional network [29].

Metal organic frameworks (MOFs) are novel porous crystalline materials composed of metal clusters and organic links that lead to a unique chemical versatility. Their highly ordered crystalline structures, large specific surface area, controllable porosity and good thermal stability make them suitable for a wide range of applications in the fields of energy storage [30,31], sensing [32], drug delivery [33], catalysis [34,35], photonics [36,37] and magnetism [38]. In recent years, the burgeoning applications of MOFs has accelerated their development and various metals, clusters and organic ligands can be selected to obtain different structural properties specific for a desired application. For example, the photocatalytic properties of MOFs can be altered by changing the distribution of metal clusters and organic ligands or by the modification of surface functional groups. This has resulted in the ability to optimize MOF formulations with improved visible light absorption [39] for photocatalytic applications. In previous reports, MOFs as photocatalysts for H_2_ production have mainly been Ti-based [40], Zr-based [41], Cr-based [42], or based on other transition metal ions [43,44,45].

Interestingly, MOFs can also act as sacrificial templates that can be converted into target multicomponent nanomaterials as active catalysts. To the best of our knowledge, no MOF-derived g-C_3_N_4_ materials as photocatalysts for hydrogen production under visible light irradiation have been reported. In this study, we developed a new strategy for the preparation of metal ion-doped g-C_3_N_4_ (M-C_3_N_4_) derived from MOFs prepared with melamine and 2,5-thiophenedicarboxylic acid, with organic solvent serving as the capping agents for modulating growth under ambient conditions; a facile hydrothermal method and subsequent calcination provides M-C_3_N_4_. We focused on the Ce-doped derivatives (Ce-C_3_N_4_) that possess a fibrous structure. Furthermore, modifications using, e.g., fluorine, can be incorporated in the MOF precursor. Ce-C_3_N_4_ modified with fluorine provides superior photocatalytic activity under visible light irradiation due to the panchromatic absorption of visible light and fine fibrous structure that imparts a high specific surface area and abundant active sites.

## 2. Materials and Methods 

### 2.1. Reagents and Materials 

All chemicals in this work were commercially available and used without any further purification. Melamine (99%), 2,5-thiophenedicarboxylic acid (98%), titanium(IV) butoxide (Ti(OC_4_H_9_)_4_, 99%), zirconyl chloride octahydrate (ZrOCl_2_·8H_2_O, AR, 99%), cerium(III) nitrate hexahydrate (Ce(NO_3_)_3_·6H_2_O, 99.5%), erbium(III) nitrate pentahydrate (Er(NO_3_)_3_·5H_2_O, 99.9%), ammonium fluoride (NH_4_F, 98%), sodium bicarbonate (NaHCO_3_, 99.8%), cellulose acetate, *N*,*N*-dimethylformamide (DMF, AR, 99.5%), triethanolamine (TEOA, AR, 98%), and sodium sulfate (Na_2_SO_4_, AR, 99%) were purchased from Aladdin, Shanghai, China.

### 2.2. Material Synthesis

#### 2.2.1. Synthesis of M-C_3_N_4_

Typically, 0.015 mol metal salt (Ti(OC_4_H_9_)_4_, ZrOCl_2_·8H_2_O, Ce(NO_3_)_3_·6H_2_O, or Er(NO_3_)_3_·5H_2_O) and 0.0025 mol melamine were dispersed in a mixture of DMF, ethanol, and water (2:3:1 v/v/v) under magnetic stirring. The as-prepared solution was then transferred to a 100 mL Teflon-lined stainless steel autoclave and heated in an oven at 130 °C for 12 h, resulting in the MOF precursor. The MOF precursor was then rinsed several times with deionized water and ethanol, dried at 50 °C for 48 h to obtain M-MOF (M = Ti^4+^, Zr^4+^, Ce^3+^, or Er^3+^), and then annealed at 550 °C for 4 h under Ar at ambient pressure before cooling to room temperature and grinding to a powder to obtain M-C_3_N_4_ (M = Ti^4+^, Zr^4+^, Ce^3+^, or Er^3+^).

#### 2.2.2. Synthesis of xCe-C_3_N_4_

Ce-C_3_N_4_ was prepared with four different amounts of Ce^3+^, denoted as xCe-C_3_N_4_ (x = 0.010, 0.015, 0.030, or 0.090 mol). The number of moles of 2,5-thiophenedicarboxylic acid and melamine were 0.007 mol and 0.0025 mol, respectively, following the procedure in Section 2.2.1, resulting in 0.010 Ce-C_3_N_4_, 0.015 Ce-C_3_N_4_, 0.030 Ce-C_3_N_4_, or 0.090 Ce-C_3_N_4_.

#### 2.2.3. Synthesis of Y-CN

Ce-C_3_N_4_ (referred to as CN) was modified using 1 wt% NH_4_F, NaHCO_3_, or cellulose acetate during preparation of the MOF precursor following the procedure in Section 2.2.1, resulting in NF-CN (NH_4_F modified Ce-C_3_N_4_), NHC-CN (NaHCO_3_ modified Ce-C_3_N_4_), and CA-CN (cellulose acetate modified Ce-C_3_N_4_), respectively.

### 2.3. Characterizations

X-ray diffraction (XRD) patterns (Bruker Co., Karlsruhe, Germany) were measured by using Bruker Model D8 Advance with Cu Kα radiation. Solid-state UV-vis (Ultraviolet–visible spectroscopy) diffuse reflectance spectra (Shimadzu Co., Kyoto, Japan) were measured using a Shimadzu UV-2600 UV–vis spectrophotometer. The photoluminescence (PL) spectra (HORIBA Ltd., Paris, France) was detected on a HORIBA Jobin Yvon LabRAM HR spectrometer at room temperature with an excitation wavelength of 365 nm. Scanning electron microscopy (SEM) images (Hitachi, Ltd., Tokyo, Japan) were obtained using a Hitachi SU-70 high-resolution scanning electron microscope.

### 2.4. Photocatalytic Measurements

Photocatalytic hydrogen production experiments were carried out in a 500 mL Pyrex glass reactor (CLE-SPH2N reaction cell, Aulight Ltd., Beijing, China) using 10 mg catalyst, 10 mL TEOA, and 100 μL 3 wt% chloroplatinic acid added to 100 mL deionized water. TEOA was used as the hole sacrificial agent to prevent oxygen production. The solution was irradiated with visible light (λ ≥ 420 nm) and the optical power density was 100 mW/cm^2^. The photocatalytic performance of the samples was evaluated by measuring the rate of hydrogen generation during the photocatalytic water splitting reaction with Gas Chromatograph 7920 (Aulight Ltd., Beijing, China).

### 2.5. Photoelectrochemical Measurement

Fluorine-doped tin oxide (FTO) (1.5 × 2 cm^2^) was used as a substrate to support samples (10 mg) that were ultrasonically dispersed in 20 mL ethanol and then added dropwise to coat the FTO. The sample loaded FTO was then dried at 60 °C for 2 h and a 1.5 × 1.5 cm^2^ sample area was used for the photoexcitation experiment.

The photocurrent was measured on a CHI 660E electrochemical workstation with a three-electrode system, in which the prepared electrode was used as the working electrode, a Pt wire (CH Instruments, Inc.) was used as the counter electrode, and a Ag/AgCl (CH Instruments, Inc.) electrode was used as the reference electrode. A 0.5 M Na_2_SO_4_ solution was used as the electrolyte. A Xe lamp (CEL-HXF300, CEAULIGHT) with AM 1.5 filter (CEL-AM 1.5, CEAULIGHT) was used as the light source. The light intensity was regulated to 50 mW·cm^−2^ by adjusting the distance between the light source and the sample.

## 3. Results

**Figure 1:**
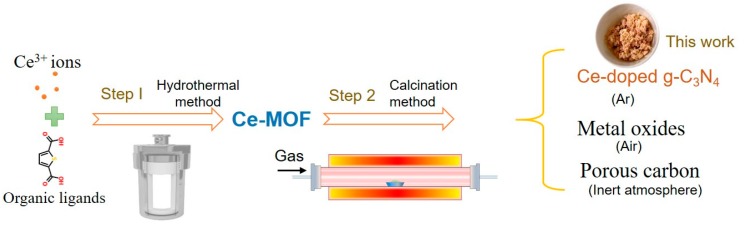
Schematic representation of the formation process of Ce-C_3_N_4_.

The process to obtain various derivatives of g-C_3_N_4_ from the MOF precursor is illustrated in Figure 1. Typically, metal coordination polymers consisting of 2,5-thiophenedicarboxylic acid and cerium(III) nitrate in the presence of melamine formed the MOF structure. Melamine is both a source of nitrogen and carbon, and also provides an alkaline environment for the reaction to proceed. After coordination polymerization to obtain the MOF precursor, gradient thermal decomposition of melamine occurs, and Ce-C_3_N_4_ was obtained by calcination under Ar at ambient pressure, forming a hierarchical network structure. This synthetic strategy can be further extended using different transition metal or lanthanide ions to obtain the MOF precursors from 2,5-thiophenedicarboxylic acid and melamine.

In order to compare the catalytic H_2_ production from M-C_3_N_4_, we used the same organic solvent to conduct metal doping of melamine using a hydrothermal method. The XRD pattern of the resulting material is shown in Figure S1. Based on the analysis of the morphology, structure, and composition, the influence of the organic solvents can be distinguished. EtOH has relative weak binding ability with metal cations, which can selectively adsorb on certain crystalline facets to form microrods and microtubes. DMF has medium binding ability with metal cations, resulting in the formation of a new crystalline phase. However, this binding ability is not strong enough to intercalate within the coordination frameworks [46]. Therefore, in this work we use a mixed solvent system to obtain optimal structures.

**Figure 2:**
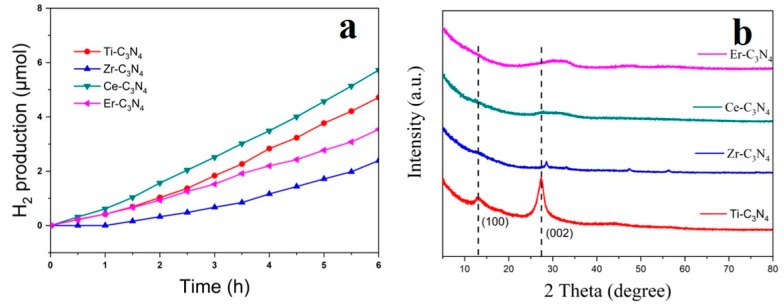
(**a**) Photocatalytic H_2_ evolution of M-C_3_N_4_ (M = Ti^4+^, Zr^4+^, Ce^3+^, or Er^3+^). (**b**) X-ray diffraction (XRD) patterns for M-C_3_N_4_ (M = Ti^4+^, Zr^4+^, Ce^3+^, or Er^3+^).

Figure 2 shows the catalytic activity of different M-C_3_N_4_ when exposed to visible light and the corresponding XRD patterns. H_2_ production of the different M-C_3_N_4_ is shown in Figure 2a. The introduction of Ce^3+^ displays the highest catalytic activity, which may be due to inhibition of crystal growth, decrease of the bandgap energy, reduced grain size, and improved separation efficiency of photoelectrons and holes [47]. The introduction of Ti^4+^ results in a higher specific surface area and larger pore volume. Ti-C_3_N_4_ can effectively extend the spectral response to the visible region [48]. Similarly, the presence of Er^3+^ dopants in samples can also increase the separation efficiency of the photogenerated electron–hole pairs [49]. From the XRD results, we can see that all the samples except for Ti-C_3_N_4_ exhibit a weak wide peak at 10–20° and 25–35°. Among these samples, Ti-C_3_N_4_ showed a significant peak corresponding to g-C_3_N_4_. Doping with the others metals affects the crystallinity of the material, mainly through formation of an amorphous phase.

**Figure 3:**
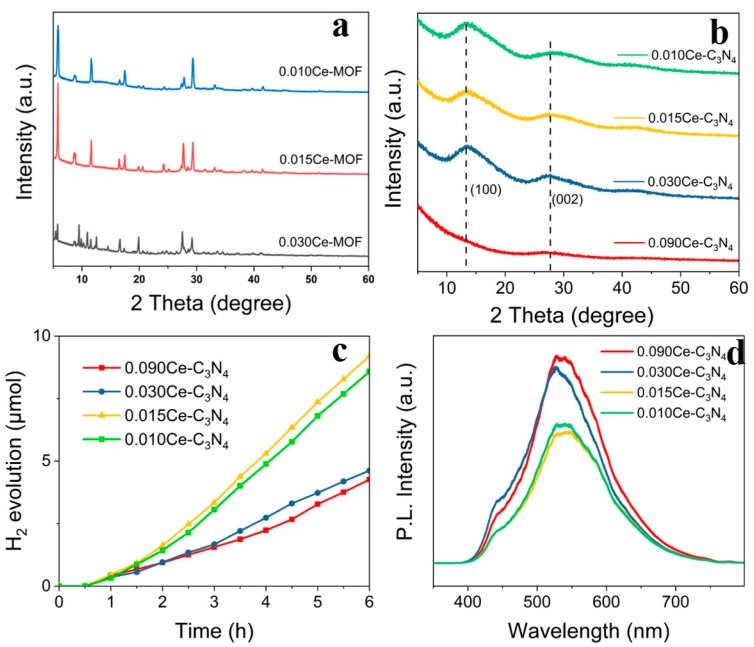
(**a**) XRD patterns for xCe-MOF (x = 0.010, 0.015, or 0.030 mol). (**b**) XRD patterns for xCe-C_3_N_4_ (x = 0.010, 0.015, 0.030, or 0.090 mol). (**c**) H_2_ evolution of xCe-C_3_N_4_ (x = 0.010, 0.015, 0.030, or 0.090 mol) as a function of time. (**d**) Photoluminescence intensity of xCe-C_3_N_4_ (x = 0.010, 0.015, 0.030, or 0.090 mol).

It can be seen from the experimental results that Ce-C_3_N_4_, after the hydrothermal method and calcination treatment, has the highest catalytic activity. Therefore, we used Ce-C_3_N_4_ derived from Ce-MOF to further explore photocatalytic H_2_ production. Figure 3a shows the XRD patterns of three xCe-MOF (x = 0.010, 0.015, or 0.030 mol), clearly showing their well-defined crystal structure. A small amount of Ce^3+^ does not significantly change the structure; however, when doping with Ce^3+^ increases, Ce^3+^ participates in the structure and it starts to change. XRD patterns indicate that most of the samples after calcination are mainly in the form of g-C_3_N_4_ (Figure 3b), in which the two peaks of the obtained samples can be fitted well to g-C_3_N_4_. The peak at 13.0° indexed as (100) indicates the in-plane packing of tri-s-triazine structures. The peak at 27.6° indexed as (002) is ascribed to the interplane stacking of the aromatic systems of g-C_3_N_4_ [50]. Most of the Ce-MOF formulations (except 0.090 mol Ce-MOF) can be converted to Ce-C_3_N_4_ by calcining under an Ar atmosphere. That the two peaks do not change among the samples implies that changing the amount of Ce^3+^ (<0.090 mol) does not influence the in-plane structure and interplanar spacing of crystals, whereas excessive introduction of Ce^3+^ also affects the formation of bonds between carbon and nitrogen in the triazine structure, resulting in an amorphous structure.

In order to demonstrate the photocatalytic activity of Ce-C_3_N_4_, H_2_ generation was monitored under visible light irradiation (Figure 3c). The order of the photocatalytic activity is as follows: 0.015Ce-C_3_N_4_ > 0.010Ce-C_3_N_4_ > 0.030Ce-C_3_N_4_ > 0.090Ce-C_3_N_4_. The 0.015Ce-C_3_N_4_ formulation shows the best photocatalytic activity among the four samples because only a small amount of Ce^3+^ doping is beneficial to improve the catalytic activity, whereas excessive doping prevents the formation of C_3_N_4_, resulting in lower activity.

PL spectra were used to analyze the recombination rate of photoexcited electrons and holes [29,30]. As shown in Figure 3d, the PL intensities of 0.015Ce-C_3_N_4_ and 0.010Ce-C_3_N_4_ were smaller than 0.090Ce-C_3_N_4_ and 0.030Ce-C_3_N_4_, indicating a lower recombination rate of photoexcited electrons and holes and longer lifetime of charge carriers, which is beneficial for photocatalytic activity. Figure S2 shows the PL results of xCe-MOF (x = 0.010, 0.015, and 0.030 mol). The intensity of 0.015Ce-MOF was lower than the other samples, suggesting that the radiative recombination of electrons and holes is more effectively inhibited, thus resulting in superior photocatalytic activity.

**Figure 4:**
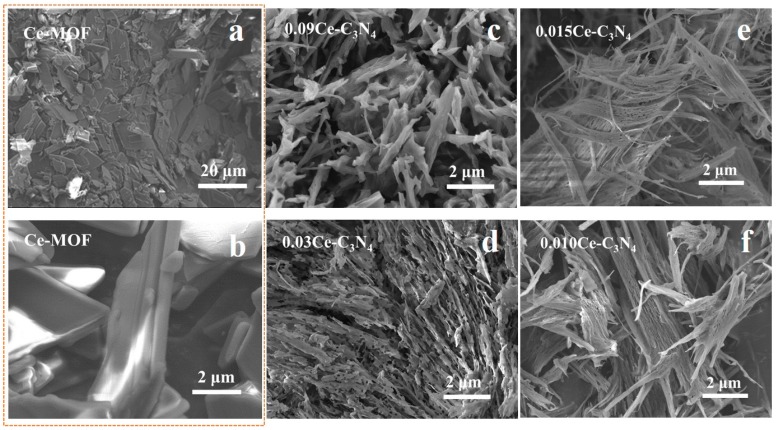
(**a,b**) SEM (Scanning Electron Microscope) images of Ce-MOF. SEM images for (**c**) 0.090Ce-C_3_N_4_, (d) 0.030Ce-C_3_N_4_, (**e**) 0.015Ce-C_3_N_4_, and (**f**) 0.010Ce-C_3_N_4_.

**Figure 5:**
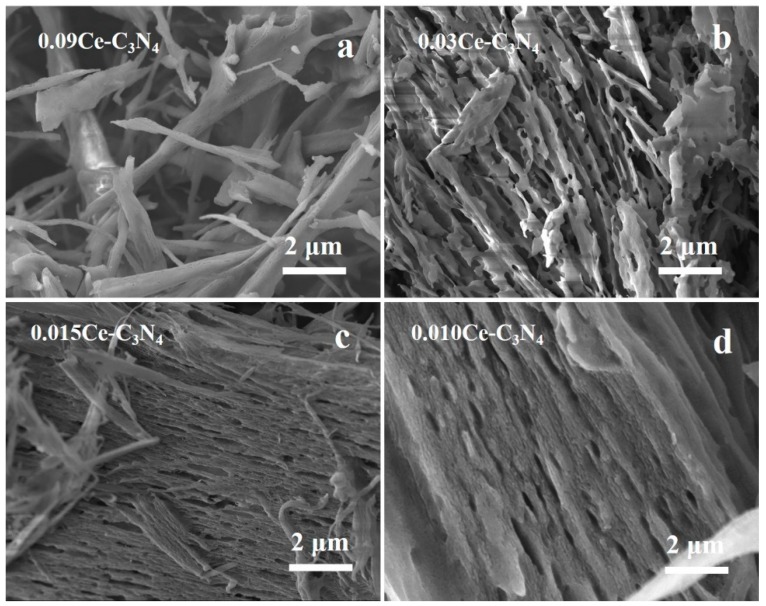
High-magnification SEM images of (**a**) 0.090Ce-C_3_N_4_, (**b**) 0.030Ce-C_3_N_4_, (**c**) 0.015Ce-C_3_N_4_, and (**d**) 0.010Ce-C_3_N_4_.

Figure 4 and Figure 5 shows the SEM images of Ce-MOF and xCe-C_3_N_4_ with various amount of Ce^3+^ (x = 0.010–0.090 mol). The unique parallelogram morphology of Ce-MOF can be observed from the low magnification and high magnification SEM images. The crystal growth of the parallelograms is along the preferred orientation epitaxy, gradually growing from a smaller nucleus into a larger crystal. This is in sharp contrast to the random block morphology before the addition of organic ligands (Figure S3). Ce-C_3_N_4_ obtained from Ce-MOF is accompanied by a transformation in the morphology as the Ce^3+^ content increases. With lower Ce^3+^ content, the morphology appears as ultra-fine fibers with strip-shaped pore structures between the fibers; with increasing Ce^3+^ content, the porous sheets resemble thick fibers lacking strip-shaped pores.

**Figure 6:**
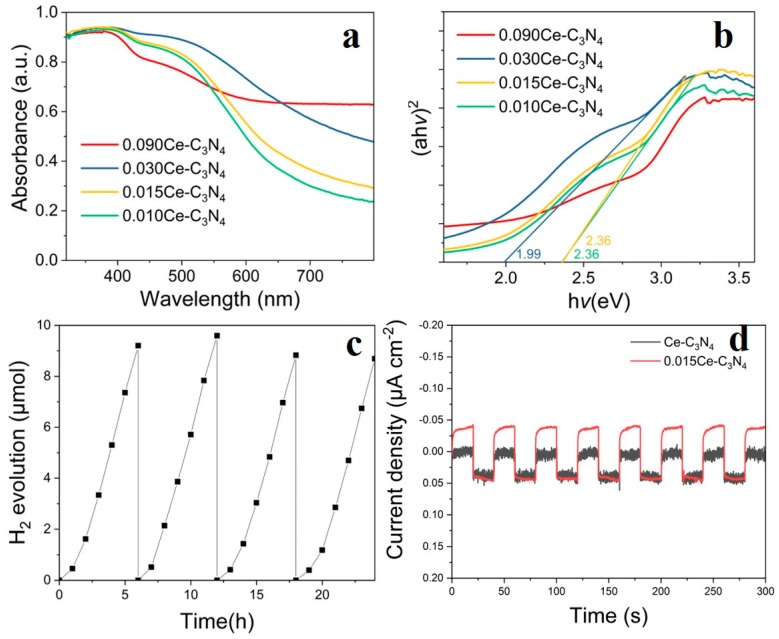
(**a**) UV-vis (Ultraviolet–visible spectroscopy) diffuse reflectance spectra of xCe-C_3_N_4_ (x = 0.010, 0.015, 0.030, and 0.090). (**b**) Plots of (αhυ)^2^ vs. photon energy (hυ) of xCe-C_3_N_4_. (x = 0.010, 0.015, 0.030, and 0.090). (**c**) The 24 h cyclic measurement of H_2_ evolution from 0.015Ce-C_3_N_4_. (**d**) Transient photocurrent response of Ce-C_3_N_4_ and 0.015Ce-C_3_N_4_.

Figure 6a,b show the UV-vis DRS, which are consistent with the photocatalytic activity. Between 400 and 800 nm, the spectra of 0.090Ce-C_3_N_4_ and 0.030Ce-C_3_N_4_ show a lower level of absorbance than 0.015Ce-C_3_N_4_ and 0.010Ce-C_3_N_4_, among which 0.015Ce-C_3_N_4_ is more redshifted than 0.010Ce-C_3_N_4_, remaining at 30% and 25% absorbance even at 800 nm, respectively, which is beneficial for optical absorption in the visible region.

The photocatalytic activity of 0.015Ce-C_3_N_4_ under visible light irradiation was performed in four cycles over 24 h (Figure 6c), revealing its excellent stability. The H_2_ production rates steadily increase during the first two cycles, decreasing slightly after three cycles. The third and fourth cycles exhibit similar H_2_ evolution efficiency, indicating that the photocatalyst is stable. This phenomenon may be due to the continuous consumption of TEOA as the reaction proceeds, with excess TEOA participating in the reflection and absorption of incident photons [51]. The H_2_ production rate is at a maximum during the second round, after which the influence of TEOA on hydrogen production gradually decreases and H_2_ production stabilizes.

The photocurrent responses of Ce-C_3_N_4_ and 0.015Ce-C_3_N_4_ are shown in Figure 6d. The mechanism of the photogenerated charge carrier transfer processes can be studied electrochemically by analyzing the transient photocurrent response [19], thus obtaining the photoelectron synthesis efficiency. The 0.015Ce-C_3_N_4_ formulation has a photocurrent response that is twice as high as that of Ce-C_3_N_4_, suggesting that the addition of organic ligands facilitates the formation of regular parallelogram structures that form fibrous g-C_3_N_4_ after calcination. The g-C_3_N_4_ material derived from the MOF may contain special surface characteristics that enable the separation efficiency of photoelectrons and holes in 0.015Ce-C_3_N_4_ to be higher than that of Ce-C_3_N_4_, therefore resulting in better photocatalytic activity.

**Figure 7:**
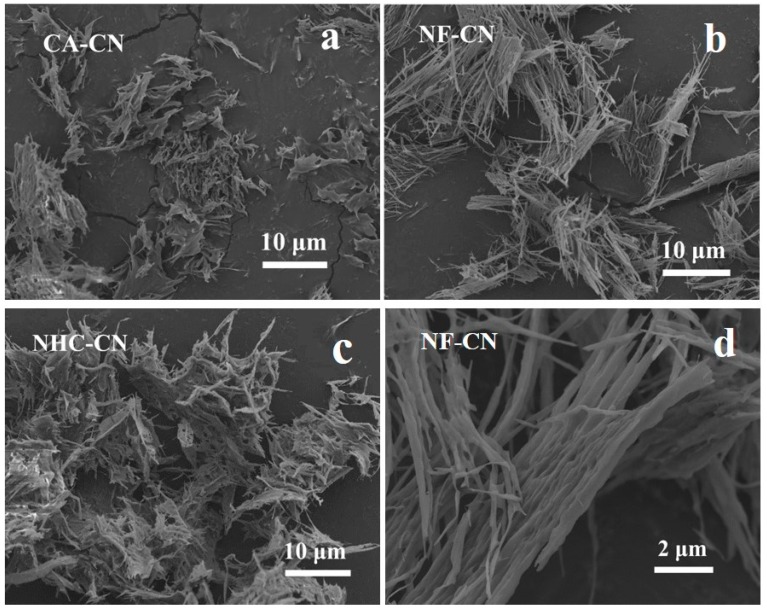
SEM images of (**a**) CA-CN (cellulose acetate modified Ce-C_3_N_4_), (**b**) NF-CN (NH4F modified Ce-C_3_N_4_), and (**c**) NHC-CN (NaHCO_3_ modified Ce-C_3_N_4_). (**d**) high-magnification SEM images of NF-CN.

In order to further optimize the performance of 0.015Ce-C_3_N_4_, different reagents were used to modify the samples. SEM images of 0.015Ce-C_3_N_4_ modified with NH_4_F, NaHCO_3_, and cellulose acetate are shown in Figure 7 and Figure S4. While the morphologies of all the modified samples changed, the type of carbon nitride did not. The NH_4_F modified sample possesses the same fibrous shape, but with finer fibers that are less stacked and more dispersed. Cellulose acetate and NaHCO_3_ modified samples both appear as mainly pleated sheets with some fibers appearing on the surface.

**Figure 8:**
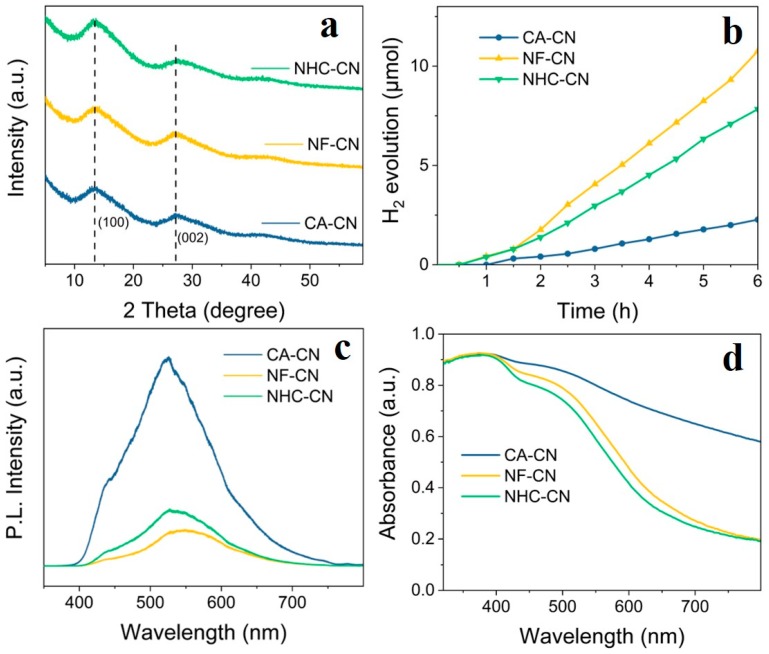
(**a**) XRD patterns of CA-CN, NF-CN, and NHC-CN. (**b**) Photocatalytic H_2_ evolution of CA-CN, NF-CN, and NHC-CN. (**c**) PL spectra for CA-CN, NF-CN, and NHC-CN. (**d**) UV-vis diffuse reflectance spectra of CA-CN, NF-CN, and NHC-CN.

XRD shows that the intense diffraction peaks at 13.3° and 27.2° of C_3_N_4_ can be observed (Figure 8a). The change in the pore structure and larger surface area facilitates surface charge interactions. The catalytic activity illustrates that the morphological structure plays a major role in the catalytic performance of the three samples (Figure 8b). Figure 8c,d indicate that the F-doped sample is photoactive under visible light illumination and can efficiently separate electrons and holes. The introduction of F facilitates uniform and defined morphology, homogeneous crystal structure, and the production of surface-bound and free hydroxyl radicals [52]. The cellulose acetate modified sample also enhances the photocatalytic activity due to its unique electronic properties, enlarged specific surface area, and improved light absorption [4]. The optical bandgap of 0.015Ce-C_3_N_4_ and NF-CN show similar bandgap values of 2.36 and 2.31 eV, respectively (Figure 6b and Figure S5). Because of the electronegativity of nitrogen and fluorine, F-doped 0.015Ce-C_3_N_4_ tends to bond with carbon instead of nitrogen, leading to the conversion of C from sp^2^ to sp^3^. The incorporation of fluorine can therefore shift both the valence band and conduction band to higher energy values [53]. Thus, doping 0.015Ce-C_3_N_4_ with fluorine forms C-F bonds and decreases the band gap from 2.36 eV to 2.31 eV, resulting in a narrower band gap and increased visible absorption.

## 4. Conclusions

In summary, we first successfully synthesized novel fibrous MOF-derived Ce-C_3_N_4_ via thermopolymerization and further thermal treatment. The optimized MOF-derived sample shows superior photocatalytic activity under visible light irradiation compared to Ce-C_3_N_4_ from cerium doped melamine. F-doped samples can be obtained upon addition of NH_4_F to the MOF precursor. NH_4_F modified samples exhibit the same fibrous shape, but with finer fibers that stack less and are more dispersed. The obtained g-C_3_N_4_ can absorb light over a wide range in the visible region. The finer fibrous structure provides abundant active sites and therefore higher photocatalytic activity. While great strides were made in this study, the preparation of hierarchical g-C_3_N_4_ with controllable doping, adjustable active sites, and structural stability still remains challenging. Although the performance of the catalyst needs to be improved, this work provides a novel strategy for designing MOF-derived photocatalysts for H_2_ energy production.

## Supplementary Materials

The following are available online at https://www.mdpi.com/2079-4991/9/11/1539/s1, Figure S1. XRD patterns of Ti-M, Zr-M, Ce-M and Er-M. Figure S2. PL spectra for xCe-MOF. (x = 0.010, 0.015, 0.030 and 0.090). Figure S3. SEM images for (**a**) Ti-C_3_N_4_, (**b**) Zr-C_3_N_4_, (**c**) Ce-C_3_N_4_, and (**d**) Er-C_3_N_4_. Figure S4. Plots of (αhυ)^2^ versus photon energy (hυ) of CA-CN, HF-CN, and NHC-CN.
