# Supplementary material for: Ce-Doped Graphitic Carbon Nitride Derived from Metal Organic Frameworks as a Visible Light-Responsive Photocatalyst for H2 Production
Source: Nanomaterials (Basel). 2019 Oct 30;9(11):1539. doi: 10.3390/nano9111539 (PMC6915336; doi:10.3390/nano9111539)
Supplement: Supplementary file 1 [file nanomaterials-09-01539-s001.pdf]

Supplementary data

Ce-Doped Graphitic Carbon Nitride Derived from Metal Organic Frameworks as a Visible Light-Responsive Photocatalyst for H_2 Production

Liangjing Zhang [1], Zhengyuan Jin [2], Shaolong Huang [2], Yiyue Zhang [2], Mei Zhang [3] and Yu-Jia Zeng [2,*], Shuangchen Ruan [1,*]

1. Center for Advanced Material Diagnostic Technology, Shenzhen Technology University, Shenzhen 518118, China; zhangliangjing@sztu.edu.cn (L.Z.)
2. Shenzhen Key Laboratory of Laser Engineering, College of Physics and Optoelectronic Engineering, Shenzhen University, Shenzhen 518060, China; zhengyuan@szu.edu.cn (Z.J.); nkhsl3313@163.com (S.H.); yiyuezhang92@163.com (Y.Z.)
3. School of Materials Science and Engineering, Beijing Institute of Fashion Technology, Beijing 100029, China. zhangmei7115@163.com (M.Z.)

* Correspondence: yjzeng@szu.edu.cn (Y.J.Z); scruan@szu.edu.cn (S.R.); Tel.: +86-0755-26532316 (Y.J.Z); +86-0755-26532350 (S.R.)

Contents

Figures

Figure S1. XRD patterns of Ti-M, Zr-M, Ce-M and Er-M.

Figure S2. PL spectra for xCe-MOF. (x = 0.010, 0.015, 0.030 and 0.090).

Figure S3. SEM images for (a) Ti-C_3N_4, (b) Zr-C_3N_4, (c) Ce-C_3N_4 and (d) Er-C_3N_4.

Figure S4. High-magnification SEM images of (a) CA-CN and (b)NHC-CN.

Figure S5 Plots of $(\alpha h\upsilon)^2$ vs. photon energy (hυ) of CA-CN, HF-CN and NHC-CN.

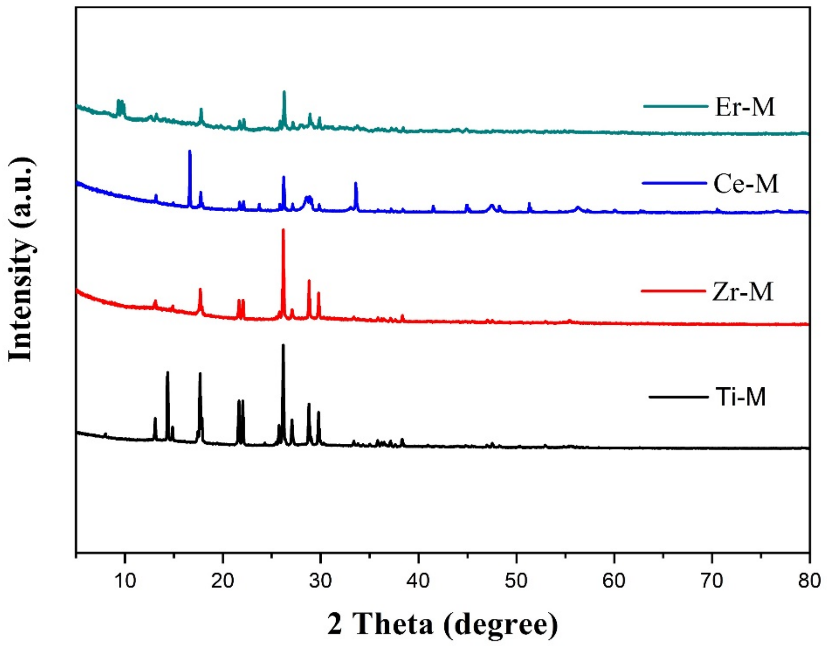

Figure S1. XRD patterns of Ti-M, Zr-M, Ce-M and Er-M.

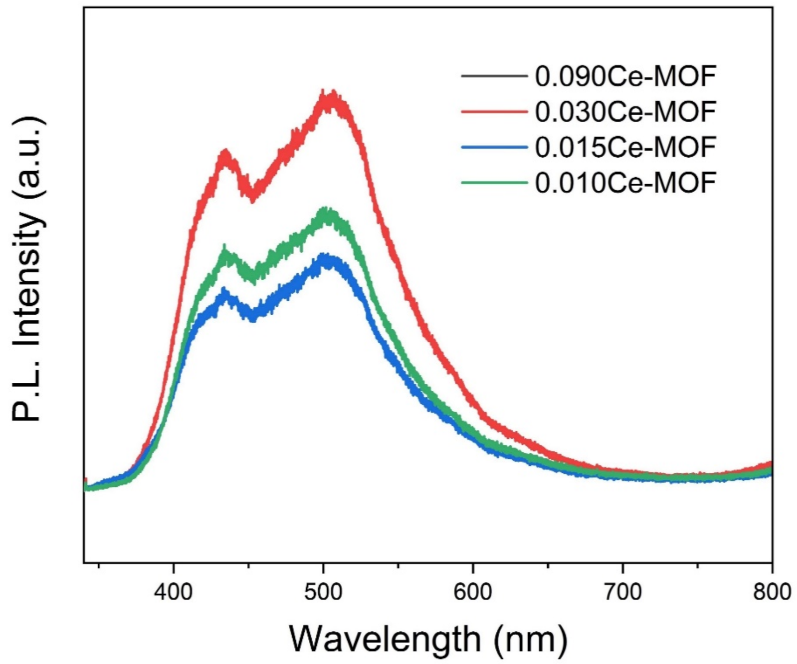

Figure S2. PL spectra for xCe-MOF. (x = 0.010, 0.015, 0.030 and 0.090).

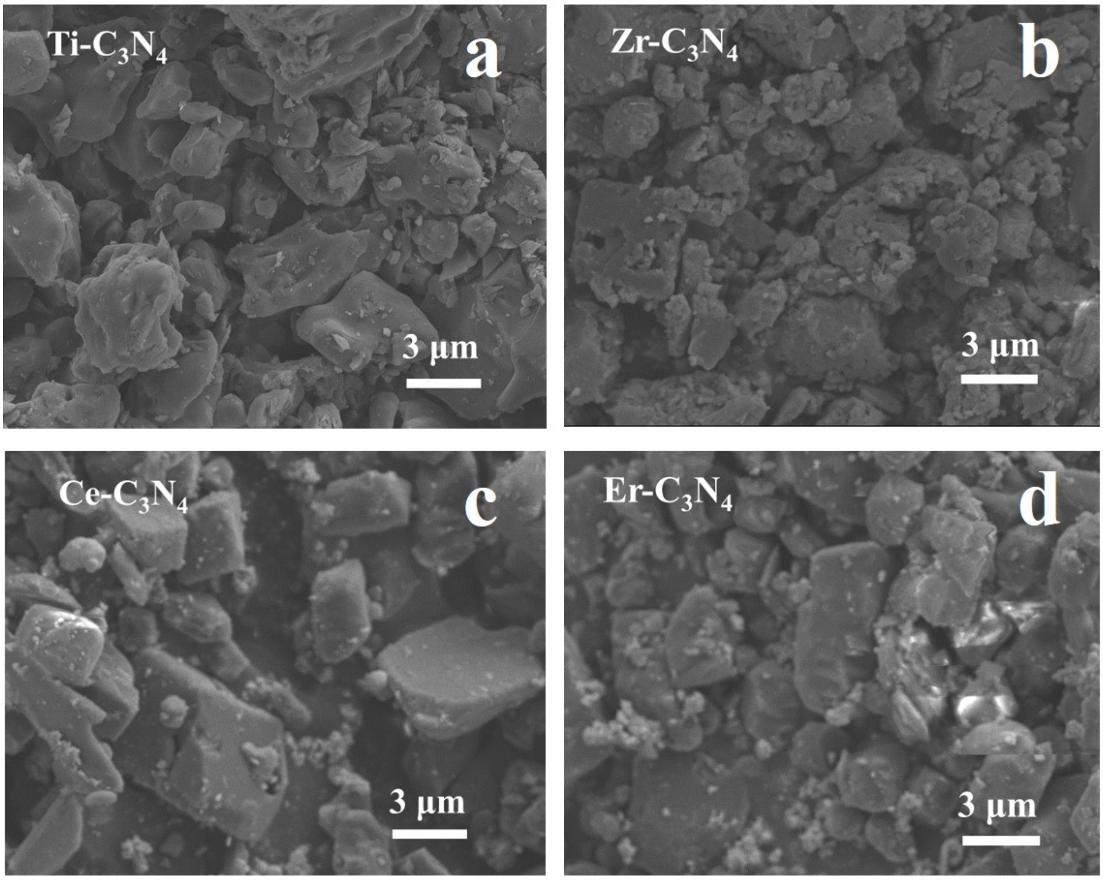

Figure S3. SEM images for (a) Ti-C_3N_4, **(b)** Zr-C_3N_4, (c) Ce-C_3N_4 and (d) Er-C_3N_4.

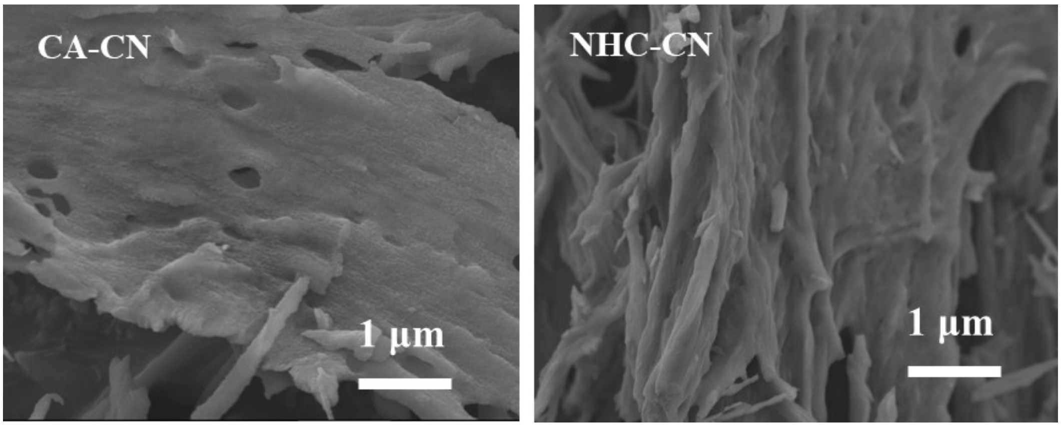

Figure S4 High-magnification SEM images of (a) CA-CN and (b)NHC-CN.

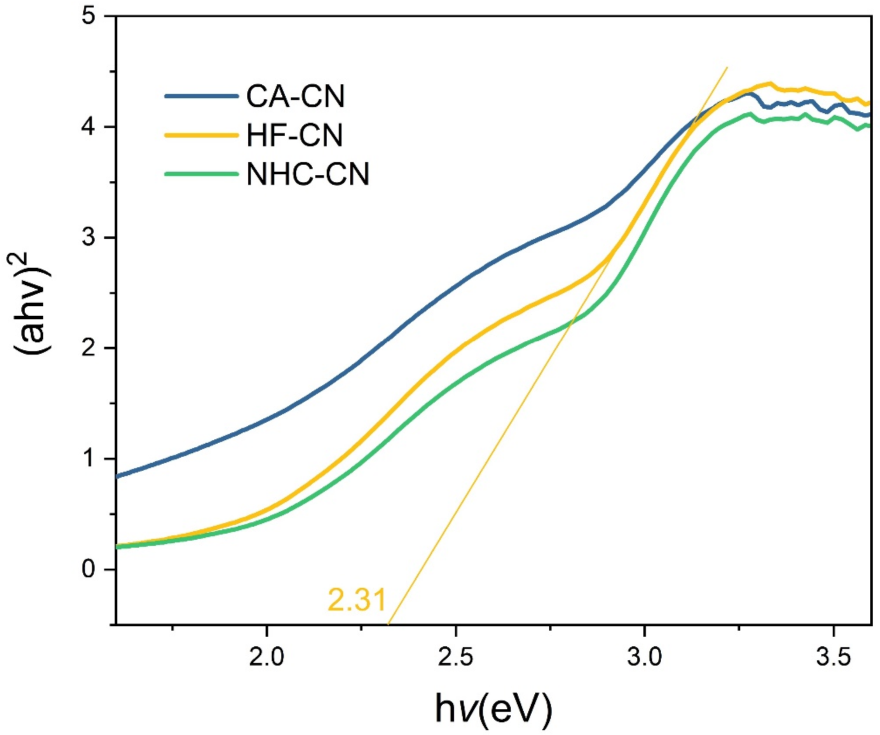

Figure S5. Plots of $(\alpha h\upsilon)^2$ vs. photon energy ($h\upsilon$) of CA-CN, HF-CN and NHC-CN.
